# Effects of Slow-Release Fertilizer on Growth, Yield, and Quality of *Ziziphus jujuba* Mill. ‘Huizao’

**DOI:** 10.3390/plants15020265

**Published:** 2026-01-15

**Authors:** Xueli Wang, Ye Yuan, Shoule Wang, Tianxiang Jiang, Dingyu Fan, Juan Jin, Ying Jin, Qing Hao, Cuiyun Wu

**Affiliations:** 1 College of Horticulture and Forestry, Tarim University, Alar 843300, China; 13468408598@163.com; 2 Research Institute of Fruit and Vegetable, Xinjiang Academy of Agricultural Sciences, Urumqi 830091, China; jujubeyuan123456@126.com (Y.Y.); jtx213666@gmail.com (T.J.); fandingyu2413@163.com (D.F.); jinjuan316@126.com (J.J.); 13299891455@163.com (Y.J.); 3 Jujube Industry Research Institute of Maigaiti County, Kashgar 844600, China; wangshoule123321@sina.com; 4 Shandong Institute of Pomology, Taian 271000, China; 5 College of Forestry, Northwest A&F University, Yangling 712100, China

**Keywords:** *Ziziphus jujuba* Mill. ‘Huizao’ (Huizao), slow-release, fruit quality, growth and development

## Abstract

Aiming at the problems of tree vigor decline and unstable fruit quality caused by soil impoverishment and easy nutrient loss in the *Ziziphus jujuba* Mill. ‘Huizao’ (Huizao) producing areas of southern Xinjiang, the application effect of bag-controlled slow-release fertilizer (BCSRF) in this region remains unclear. In this study, a field experiment was conducted with four fertilization concentration gradients, including CK (0 kg/ha), T1 (22 kg/ha), T2 (44 kg/ha), and T3 (66 kg/ha), to investigate the effects of BCSRF on soil nutrient dynamics and plant growth, as well as the fruit yield and quality of Huizao. The results showed that BCSRF could effectively maintain the supply levels of soil alkali-hydrolysable nitrogen, available phosphorus, and available potassium during key growth periods, among which the T3 treatment exhibited the most significant effect. This treatment not only significantly increased the yield per plant of Huizao by 39.34% compared with the control, but also markedly enhanced the contents of the endogenous substance, including soluble sugar and cyclic adenosine monophosphate. This study confirms that under the condition of sandy loam soil in southern Xinjiang, a single basal application of an appropriate amount of BCSRF can achieve continuous nutrient supply, simultaneously improve soil fertility and fruit quality, providing a theoretical basis and technical guidance for simplified and efficient fertilization in local jujube orchards.

## 1. Introduction

*Ziziphus jujuba* Mill. (Chinese jujube), a member of the Rhamnaceae family and genus *Ziziphus*, exhibits notable tolerance to drought, saline–alkaline stress, and nutrient-poor soils [1,2,3]. Furthermore, its fruits are rich in nutritional components and possess inherent medicinal properties [4,5], rendering them highly popular in China. Notably, their nutritional profile encompasses triterpenoids, polysaccharides, cyclic nucleotides, polyphenols, flavonoids, and other bioactive compounds [6,7]. Jujube has a cultivation history of more than 4000 years in China. At present, the cultivation area exceeds 3 million hectares, with an annual output of over 7 million tons, accounting for about 98% of the global total output. As the most important jujube producing area in China, Xinjiang ranks among the top in the world in terms of output. Among them, the planting area and output in southern Xinjiang account for more than 98% of the whole of Xinjiang, making it the core jujube planting area and the national high-quality dried jujube production base in China [8,9,10]. This region is endowed with unique light and heat resources, with a significant temperature difference between day and night. Among the local cultivated varieties, the jujube variety *Ziziphus jujuba* Mill. ‘Huizao’ (hereafter Huizao) is the main high-quality jujube variety cultivated in southern Xinjiang. It has firm flesh, rich flavor, and a high sugar content, and has both fresh-eating and drying value, accounting for the largest proportion of the total output of jujubes in southern Xinjiang [11]. However, this variety generally suffers from the problem of inconsistent fruit quality. Jujube orchards in southern Xinjiang are mostly distributed on the edge of deserts, with sandy soil and sandy loam as the main soil types, which are characterized by low organic matter content and poor natural fertility. In addition, the low annual precipitation and high evaporation capacity lead to severe soil nutrient loss. These factors jointly result in the weakening of jujube tree vigor, the reduction in stress resistance, the deterioration of fruit quality, and the high incidence of diseases and insect pests, which severely restrict the sustainable development of the local jujube industry [12,13].

Slow/controlled release fertilizers (SRFs/CRFs) can effectively coordinate nutrient supply, increase soil nutrient content, and promote crop growth. Their effects of increasing yield and improving quality have been confirmed in a variety of crops. For example, controlled release fertilizers (CRF) can increase rice yield and reduce ammonia emissions [14]. Compared with farmers’ fertilization practice (FFP), under the condition of reducing nitrogen application by 16.7%, the CRFDP treatment significantly increased rice yield by 26.1–33.3% by synchronously increasing the number of effective panicles and the number of grains per panicle [15]. The treatment of replacing 15% of the nitrogen with slow-release fertilizer significantly increased soil pH value and organic matter content, while reducing the concentrations of available phosphorus (AP), available potassium (AK), nitrate nitrogen, and ammonium nitrogen [16]. Compared with the ZnSO_4_^−^ treatment, the novel slow-release zinc fertilizer (Zn-NCDs) increased the rhizome biomass, number of effective panicles, and grain yield of Sirvan by 20%, 44%, 16%, and 43%, respectively, while reducing the phytic acid content by 18% [17].

Bag-controlled slow-release fertilizer (BCSRF) is a novel slow-release fertilizer that has been increasingly adopted in China. Unlike conventional coated granular slow-release fertilizers, its controlled-release bag design modulates nutrient release to accommodate the nutrient demands of large-sized fruit trees, thereby meeting the diverse nutrient requirements of plants across growth stages and facilitating precise release [18,19]. BCSRF application has demonstrated consistent effects across agricultural systems, including fruit trees (e.g., peaches [20], grapes [21]), and field crops (e.g., maize [22], rice [23], sugar beets [24]): it sustains a stable nutrient supply, improves fertilizer use efficiency (FUE), promotes fine root growth and nutrient uptake, regulates vegetative growth, and enhances fruit/crop yield and quality. However, research on the BCSRF application in jujube cultivation remains limited. Only Peng et al. have conducted experiments in Shandong Province, China, finding that the BCSRF2 treatment significantly increased soil nutrient content, yield, and fruit quality of *Ziziphus jujuba* Mill. ‘Zhanhua’ [19]. The effects of BCSRF application in major jujube-producing regions and its impact on Huizao, the most widely cultivated jujube cultivar, remain undetermined. Therefore, to determine the optimal BCSRF application rate for Huizao grown in sandy loam soils of southern Xinjiang and to provide a theoretical basis and practical guidance for scientific fertilization, quality improvement, and yield-efficiency enhancement of local jujube trees, this study established different BCSRF application rate treatments to investigate their effects on soil nutrients in Huizao orchards, tree growth, and fruit yield and quality.

## 2. Results

### 2.1. Changes in Soil Nutrients After Application of BCSRF

**Figure 1:**
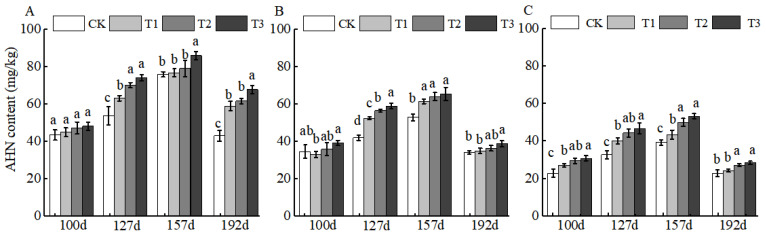
BCRSF’s impact on soil AHN nutrient content variation. Note: AHN content—alkali-hydrolysable nitrogen content. (**A**) Impact variations in BCRSF on the 0–20 cm soil layer of AHN. (**B**) Impact variations in BCRSF on the 20–40 cm soil layer of AHN. (**C**) Impact variations in BCRSF on the 40–60 cm soil layer of AHN. Data represent the mean ± standard deviation (SD) of three independent biological replicates (n = 3). Error bars indicate SD. Different lowercase letters in the same column indicate a significant difference between treatments (*p* < 0.05).

BCSRF-induced AHN release exhibited distinct depth-dependent temporal patterns. In the 0–20 cm layer, the most pronounced slow-release effect was observed at 192 d after application, with AHN increases ranging from 36.70% to 57.38% relative to the control (CK). Among treatments, T3 was the most effective, inducing a significant 57.38% increase compared to CK (Figure 1A). In contrast, in the 20–40 cm and 40–60 cm layers, peak AHN release occurred earlier at 127 d, with increases ranging from 24.54% to 40.08% (20–40 cm) and 23.02% to 43.11% (40–60 cm). Consistently, T3 remained the optimal treatment in deeper layers, with significant increases of 40.08% (20–40 cm) and 43.11% (40–60 cm) relative to CK (Figure 1B,C).

**Figure 2:**
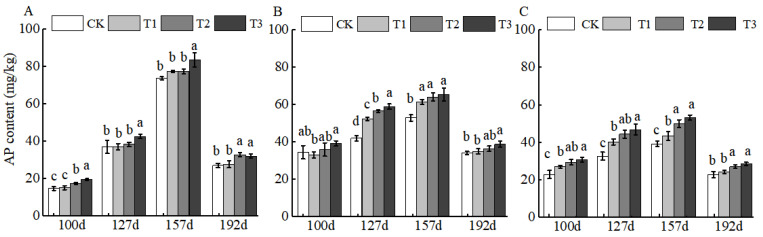
BCRSF’s impact on soil AP nutrient content variation. Note: AP content—available phosphorus content. (**A**) Impact variations in BCRSF on the 0–20 cm soil layer of AP. (**B**) Impact variations in BCRSF on the 20–40 cm soil layer of AP. (**C**) Impact variations in BCRSF on the 40–60 cm soil layer of AP. Data represent the mean ± standard deviation (SD) of three independent biological replicates (n = 3). Error bars indicate SD. Different lowercase letters in the same column indicate a significant difference between treatments (*p* < 0.05).

For AP, the primary plant-available phosphorus form, BCSRF application resulted in the most significant nutrient release at 100 d across all soil layers, with the 0–20 cm layer exhibiting the highest increase (up to 32.23% relative to CK; (Figure 2A). This early peak in AP release suggests BCSRF may efficiently support phosphorus demand during the initial growth stages of jujube trees.

**Figure 3:**
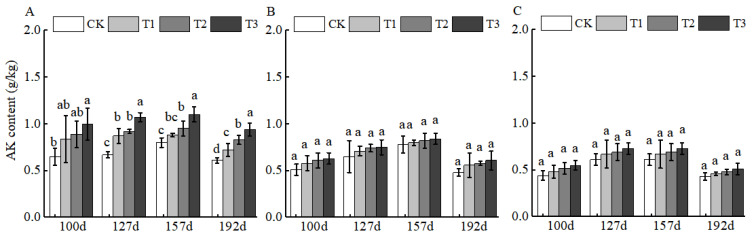
BCRSF’s impact on soil AK nutrient content variation. Note: AK content—available potassium content. (**A**) Impact variations in BCRSF on the 0–20 cm soil layer of AK. (**B**) Impact variations in BCRSF on the 20–40 cm soil layer of AK. (**C**) Impact variations in BCRSF on the 40–60 cm soil layer of AK. Data represent the mean ± standard deviation (SD) of three independent biological replicates (n = 3). Error bars indicate SD. Different lowercase letters in the same column indicate a significant difference between treatments (*p* < 0.05).

BCSRF, characterized by a high potassium content, demonstrated the most pronounced slow-release effect in the 0–20 cm layer at 127 d, with increases reaching 59.70% relative to CK (Figure 3A). However, no significant differences in AK release were observed among the four sampling time points in the 20–40 cm and 40–60 cm layers (Figure 3B,C), indicating limited downward mobility of BCSRF-derived potassium in sandy loam.

**Figure 4:**
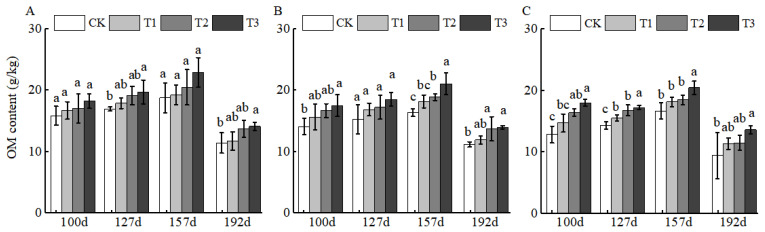
BCRSF’s impact on soil OM nutrient content variation. Note: OM content—organic matter content. (**A**) Impact variations in BCRSF on the 0–20 cm soil layer of OM. (**B**) Impact variations in BCRSF on the 20–40 cm soil layer of OM. (**C**) Impact variations in BCRSF on the 40–60 cm soil layer of OM. Data represent the mean ± standard deviation (SD) of three independent biological replicates (n = 3). Error bars indicate SD. Different lowercase letters in the same column indicate a significant difference between treatments (*p* < 0.05).

In this study, OM release exhibited phased dynamics across sampling points: from 100 to 127 d, slight OM release was detected in all soil layers, with initial treatment differences emerging. By 157 d, OM release peaked in all layers, with T3 inducing the most significant increases (32.79% in 0–20 cm, 28.63% in 20–40 cm, and 22.57% in 40–60 cm relative to CK) (Figure 4). By 192 d, OM release stabilized and declined, suggesting BCSRF has the potential to enhance soil quality in this region.

### 2.2. Changes in Growth and Yield of Huizao After Application of BCSRF

To further explore the comprehensive effects of different fertilization treatments on the growth of Huizao plants, this study systematically observed the phenological periods and the growth and development status of various organs of Huizao in each treatment group. The results showed that there were no significant differences among different treatment groups in phenological stages including bud burst period, leaf expanding period, initial flowering period, full flowering period, final flowering period, fruit white ripening period (WR), fruit crisp ripening period (CR), fruit full ripening period (FR), and leaf fall period. The annual growth cycle (207 days) and fruit development period (157 days) were consistent across all treatment groups (CK, T1, T2, T3).

Huizao produces a large number of flowers, but it has a high flower abscission rate and low fruit setting rate [25]. We found that the application of BCSRF could significantly increase the fruit setting rate and yield per plant of Huizao. Compared with CK, the fruit setting rates of T1, T2, and T3 treatments were significantly increased by 17.69%, 19.49%, and 23.67%, respectively, and the yields per plant of T1, T2, and T3 treatments were significantly increased by 15.21%, 17.31%, and 39.34%, respectively (Table 1).

**Figure 5:**
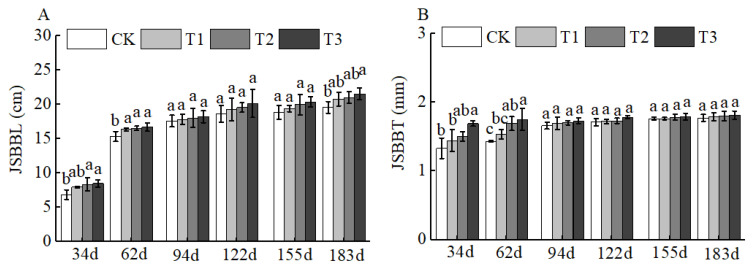
BCRSF’s impact on short bearing branches variation. Note: JSBBL—short bearing branches length. JSBBT—short bearing branches thickness. (**A**) Impact variations in BCRSF on the short bearing branches’ length. (**B**) Impact variations in BCRSF on the short bearing branches’ thickness. Data represent the mean ± standard deviation (SD) of three independent biological replicates (n = 3). Error bars indicate SD. Different lowercase letters in the same column indicate a significant difference between treatments (*p* < 0.05).

**Figure 6:**
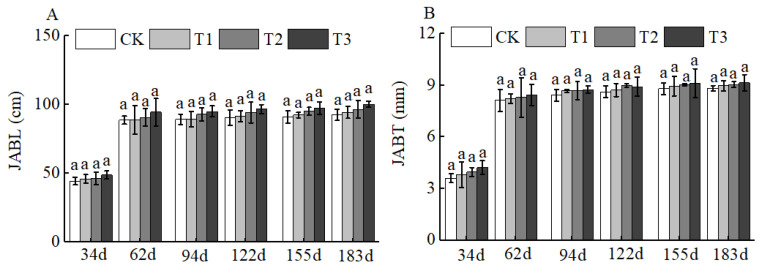
BCRSF’s impact on annual branch variation. Note: JABL—annual branch length. JABT—annual branch thickness. (**A**) Impact variations in BCRSF on the annual branch length. (**B**) Impact variations in BCRSF on the annual branch thickness. Data represent the mean ± standard deviation (SD) of three independent biological replicates (n = 3). Error bars indicate SD. Different lowercase letters in the same column indicate a significant difference between treatments (*p* < 0.05).

**Figure 7:**
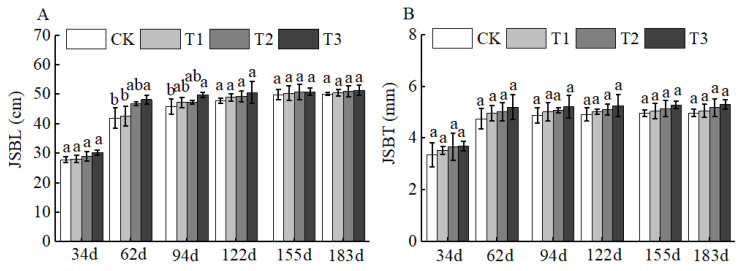
BCRSF’s impact on secondary branches variation. Note: JSBL—secondary branches length. JSBT—secondary branches thickness. (**A**) Impact variations in BCRSF on the secondary branches’ length. (**B**) Impact variations in BCRSF on the secondary branches’ thickness. Data represent the mean ± standard deviation (SD) of three independent biological replicates (n = 3). Error bars indicate SD. Different lowercase letters in the same column indicate a significant difference between treatments (*p* < 0.05).

**Figure 8:**
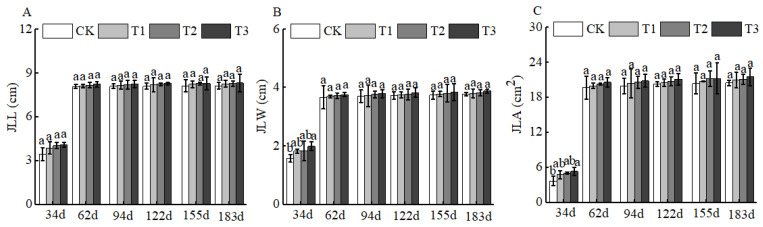
BCRSF’s impact on leaf variation. Note: JLL—leaf length. JLW—leaf width. JLA—leaf area. (**A**) Impact of variations in BCRSF on the leaf length. (**B**) Impact variations in BCRSF on the leaf width. (**C**) Impact variations in BCRSF on the leaf area. Data represent the mean ± standard deviation (SD) of three independent biological replicates (n = 3). Error bars indicate SD. Different lowercase letters in the same column indicate a significant difference between treatments (*p* < 0.05).

Of course, BCSRF could not affect the apparent traits of floral organs, but it could influence the growth of short bearing branches, secondary branches, annual branches, and leaves of Huizao to a certain extent. At 34 d after BCSRF application, its promoting effect on the growth of short bearing branches and leaves was the most concentrated and significant, showing a trend of the promoting effect enhancing with the increase in fertilizer application rate. Compared with CK, the T1, T2, and T3 treatments significantly increased the length and thickness of short bearing branches, leaf width, and leaf area (Figure 5). The T3 treatment exhibited the most significant effect: the length and thickness of the short bearing branch in T3 were significantly increased by 23.79% and 27.06% compared with CK, respectively, while the leaf width and leaf area were significantly increased by 25.95% and 38.15% compared with CK, respectively *(p* < 0.05). At 62 d, 94 d, and 183 d after BCSRF application, the fertilizer only promoted the growth of short bearing branches and the length of secondary branches, and the promoting effect was less significant than that at 34 d after application (Figure 5, Figure 6 and Figure 7). Among different treatments, the T3 treatment was the most effective: At 62 d after application, the thickness of the short bearing branch in T3 was significantly increased by 22.38% compared with CK, and the length of secondary branches in T3 was significantly increased by 14.85% compared with CK; at 94 d after application, only the growth of secondary branch length was promoted, and the length in T3 was significantly increased by 8.50% compared with CK; at 183 d after application, only the length of the short bearing branch was promoted, and the length in T3 was significantly increased by 10.13% compared with CK (*p* < 0.05) (Figure 7 and Figure 8).

### 2.3. Changes in Weight, Shape Dimensions, and Nutritional Quality of Huizao After Application of BCSRF

After the application of BCSRF, during the fruit development period, there were significant differences in the single fruit weight of Huizao between the T1, T2, and T3 treatments and CK at the white ripening period and full ripening period (Table 2). Specifically, compared with CK, the single fruit weight in T1, T2, and T3 treatments was significantly increased by 2.62%, 8.91%, and 27.33% (at the white ripening period) and 2.38%, 9.93%, and 14.61% (at the full ripening period), respectively (*p* < 0.05). However, transverse thickness and fruit shape index showed no significant effect on Huizao at different ripening stages.

**Figure 9:**
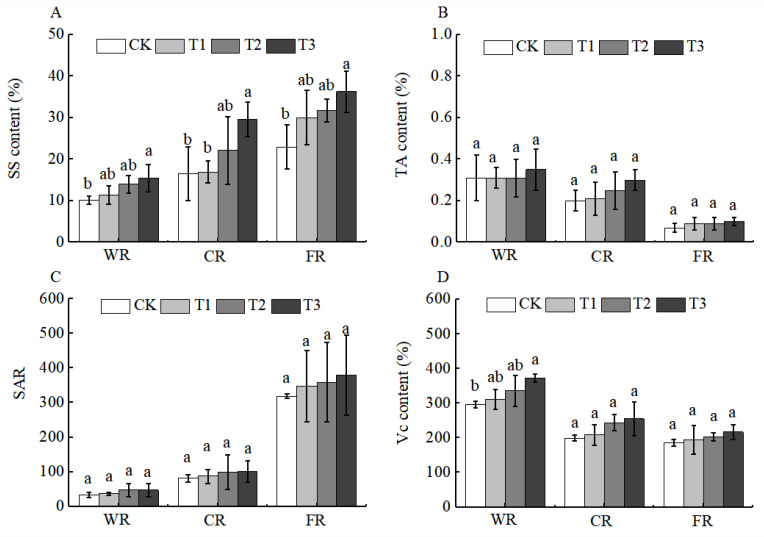
BCRSF’s impact on fruit quality variation. Note: SS—soluble sugars. TA—titratable acids. SAR—sugar-acid ratio. Vc—vitamin C. (**A**) Impact variations in BCRSF on the soluble sugars. (**B**) Impact variations in BCRSF on the titratable acids. (**C**) Impact variations in BCRSF on the sugar-acid ratio. (**D**) Impact variations in BCRSF on the vitamin C. Data represent the mean ± standard deviation (SD) of three independent biological replicates (n = 3). Error bars indicate SD. Different lowercase letters in the same column indicate a significant difference between treatments (*p* < 0.05).

Fruit nutritional quality determines taste and flavor. Basic nutritional components such as sugars and acids in fruits are not only important indicators for evaluating fruit quality but also crucial carriers of fruit nutrients [26]. After the application of BCSRF, the contents of soluble sugars (SS) and titratable acids (TA) were affected to a certain extent during the fruit development period, among which soluble sugars were the most significantly affected. The content of SS showed an upward trend with the ripening of Huizao fruits, and this trend began at the white ripening period. At the full ripening period, the SS content reached the highest level, with the T3 treatment being the most significant, showing a significant increase of 58.08% compared with CK (Figure 9A). This may be related to the fact that the accumulation of SS depends on photosynthates synthesized by leaf photosynthesis. Although the content of TA and the sugar–acid ratio changed to some extent, there was no significant difference among treatments (Figure 9B,C).

**Figure 10:**
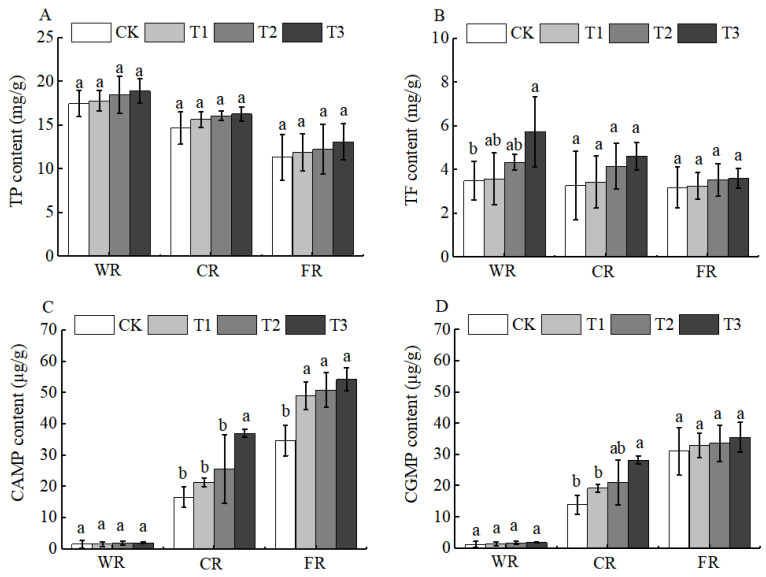
BCRSF’s impact on fruit quality variation. Note: TP—total phenols. TF—total flavonoids. CAMP—cyclic adenosine monophosphate. CGMP—cyclic guanosine monophosphate. (**A**) Impact of variations in BCRSF on the total phenols. (**B**) Impact variations in BCRSF on the total flavonoids. (**C**) Impact variations in BCRSF on the cyclic adenosine monophosphate. (**D**) Impact variations in BCRSF on the cyclic guanosine monophosphate. Data represent the mean ± standard deviation (SD) of three independent biological replicates (n = 3). Error bars indicate SD. Different lowercase letters in the same column indicate a significant difference between treatments (*p* < 0.05).

Jujube fruits are also rich in functional components such as vitamin C (Vc), total flavonoids (TF), total phenols (TP), cyclic adenosine monophosphate (cAMP), and cyclic guanosine monophosphate (cGMP). These components form a “functional complementarity” and collectively enhance the health value of jujubes [27]. After the application of BCSRF, the contents of functional components, including Vc, TF, and TP, showed a downward trend during fruit development (white ripening period, crisp ripening period, and full ripening period), and there were different changes among different treatments compared with CK. Specifically, the contents of Vc and TF reached the highest level at the white ripening period, with significant differences among treatments. The T3 treatment was the most significant: compared with CK, the contents of Vc and TF in T3 were significantly increased by 25.84% and 64.00%, respectively (Figure 9D and Figure 10A).

Although the content of TP changed to a certain extent, there was no significant difference among treatments. In contrast, the contents of cAMP and cGMP showed an upward trend during fruit development (white ripening period, crisp ripening period, and full ripening period), and different treatments exhibited different changes compared with CK during the fruit development period. At the crisp ripening period, the contents of cAMP and cGMP in each treatment showed significant differences compared with CK. Among them, the T3 treatment had the best effect, with significant increases of 122.41% and 101.06% in cAMP and cGMP contents compared with CK, respectively (*p* < 0.05). At the full ripening period, the cAMP content in the T3 treatment was significantly increased by 56.11% compared with CK, but there was no significant difference in cGMP content between any treatment and CK (*p* < 0.05) (Figure 10B–D).

### 2.4. Changes in Photosynthesis of Huizao After Application of BCSRF

**Figure 11:**
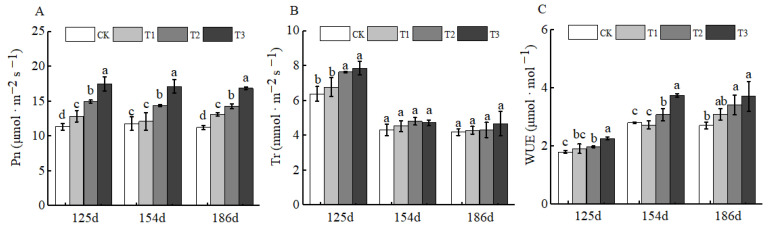
BCRSF’s impact on photosynthesis variation. Note: Pn—net photosynthetic rate. Tr—transpiration rate. WUE—water use efficiency. (**A**) Impact variations in BCRSF on the net photosynthetic rate. (**B**) Impact variations in BCRSF on the transpiration rate. (**C**) Impact variations in BCRSF on the water use efficiency. Data represent the mean ± standard deviation (SD) of three independent biological replicates (n = 3). Error bars indicate SD. Different lowercase letters in the same column indicate a significant difference between treatments (*p* < 0.05).

Fertilizers play a crucial role in plant growth and development, particularly in plant photosynthesis. As the foundation of plant growth and development, photosynthesis is also one of the key processes for organic matter accumulation in plants [28]. After the application of BCSRF, the net photosynthetic rate (Pn) and transpiration rate (Tr) showed a downward trend. However, at 125 d, 154 d, and 186 d after BCSRF application, the Pn values of T1, T2, and T3 treatments were significantly higher than those of CK, with the T3 treatment showing the most prominent advantage (Figure 11A,B). Specifically, compared with CK, the Pn of T3 was significantly increased by 54.13%, 45.21%, and 50.22% at the three time points, respectively (*p* < 0.05). This may be related to the fact that BCSRF maintains a relatively high net photosynthetic rate by continuously supplying nutrients, thereby providing a stable carbon source for fruit quality formation. The water use efficiency (WUE) showed an upward trend. At 125 d, 154 d, and 186 d after BCSRF application, the WUE of the BCSRF-treated groups was significantly higher than that of CK. Among them, the WUE of the T3 treatment at 186 d after BCSRF application was significantly increased by 36.90% compared with CK, which was the result of the synergistic effect of decreased Pn and Tr (Figure 11C).

**Figure 12:**
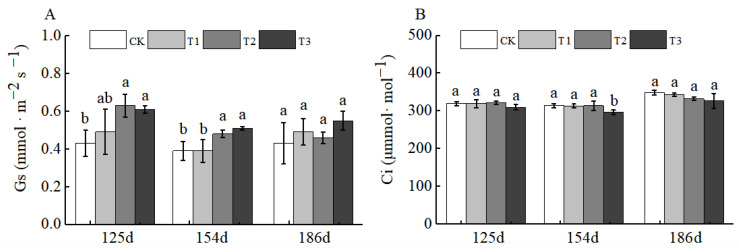
BCRSF’s impact on photosynthesis variation. Note: Gs—stomatal conductance. Ci—intercellular carbon dioxide concentration. (**A**) Impact variations in BCRSF on the stomatal conductance. (**B**) Impact variations in BCRSF on the intercellular carbon dioxide concentration. Data represent the mean ± standard deviation (SD) of three independent biological replicates (n = 3). Error bars indicate SD. Different lowercase letters in the same column indicate a significant difference between treatments (*p* < 0.05).

No obvious change trends were observed for stomatal conductance (Gs) and intercellular carbon dioxide concentration (Ci). For Gs, only the T1, T2, and T3 treatments at 125 d after BCSRF application showed a significant increase compared with CK. For Ci, there was no significant difference between any BCSRF treatment and CK at 125 d, 154 d, and 186 d after application (Figure 12A,B).

## 3. Discussion

Fertilization directly affects soil nutrient contents and its productivity. Excessive application of chemical fertilizers will impair soil composition as well as its properties such as fertility and integrity [29,30]. Available nitrogen (AHN) and available potassium (AK) in soil are key available nutrients that directly participate in the nutrient cycling of the plant–soil system, and both play an irreplaceable role in maintaining soil fertility for the sustainable management of orchards [31,32,33]. Other studies have demonstrated that in the soil of jujube orchards applied with conventional fertilizers, the contents of AHN, AP, and AK show a decreasing trend with the deepening of soil layers. In addition, insufficient nutrient supply during the middle and late stages of growth fails to meet the high nutritional requirements of fruit trees in this period, which results in poor fruit quality and low yield and ultimately exerts an adverse impact on the growth of Huizao [34]. The application of BCSRF in this study effectively regulated the dynamics of soil nutrient supply. After the application of BCSRF, different release durations exhibited differential slow-release effects on the contents of AHN, AP, and AK in different soil layers of jujube orchards in southern Xinjiang. Particularly during the critical periods of fruit ripening and sugar accumulation (e.g., at 157 and 192 days after BCSRF application), the contents of AHN and AK in the soil were maintained at high levels (Figure 1 and Figure 3), which satisfied the vigorous physiological demands of fruit trees, prolonged the duration of soil nutrient supply, and enhanced the soil fertility supply capacity during the late growth stage of Huizao. This is consistent with the characteristic of slow-release fertilizers (SRF) that they can improve soil fertility and plant nutrient uptake capacity, and slowly supply nutrients according to the different growth stages of plants [35,36,37]. It also provides a reference for precise nutrient management to achieve high-yield and high-quality jujube cultivation under the special site condition of sandy loam soil in the desert oases of southern Xinjiang. In terms of the comparison of nutrient effects of different slow-release fertilizers on different soil types, all of them can increase soil nutrient contents [38,39]. Meanwhile, OM is a core indicator for evaluating soil quality and fertility, and it directly determines the capacity of soil to support crop growth [40]. In terms of OM content, the T3 treatment achieved the peak OM content in the 0–20 cm soil layer at 157 days after fertilization (Figure 4A). This indicates that BCSRF can not only increase soil nutrients but also contribute to the accumulation and maintenance of OM stability, which is of great significance for improving the physical and chemical properties of sandy loam soil in southern Xinjiang. In comparison with the effects of other slow-release fertilizers on different soil types, the slow-release granular fertilizer prepared by mixing lignite and urea (BCU) showed that its fertilizer efficiency and environmental benefits depended on soil types and their physical and chemical properties through laboratory soil column incubation experiments, which significantly affected the soil clay content and organic matter level [41].

During the fertilization process, different application timings and concentrations are particularly critical for the accumulation and translocation of fruit quality attributes. At present, there are few reports on the effects of slow-release fertilizers on jujube fruit quality, whereas relevant studies regarding the impacts of fertilization per se have been documented. Compared with other treatments, the application of slow-release fertilizer under the BCRF2 regime significantly increased the single fruit weight and soluble sugar content of *Ziziphus jujuba* Mill. ‘Zhanhua’ [19]. In this study, the T3 treatment promoted increases in the single fruit weight and soluble sugar content of Huizao at the full ripening stage (Table 2, Figure 9A). This finding is consistent with the aforementioned study. It is worth noting that the same effects can also be achieved with variations in fertilizer application methods, application concentrations, tree ages, and jujube cultivars, indicating that the efficacy of slow-release fertilizers in improving jujube fruit quality has certain universality and applicability. However, research on the mechanisms underlying the effects of slow-release fertilizers on the contents of functional components such as Vc, TP, cAMP, and cGMP in jujube fruits remains relatively limited. This study found that the BCSRF treatment, especially the T3 treatment, significantly increased the contents of Vc, TP, cAMP, and cGMP in Huizao fruits (Figure 9D and Figure 10), particularly during the CR and FR of jujube fruits. This finding is consistent with the results of studies on the effects of slow-release fertilizers on functional components in other plants. Compared with the TF treatment, both slow-release fertilizer (SRF) and double-coated slow-release fertilizer (DSRF) treatments significantly increased the flavonoid content in Chinese chives (*Allium tuberm* Rottler ex Spreng.) in greenhouses during winter, with respective increases of 16% and 19% [42]. Specifically formulated slow-release fertilizer treatments can also substantially enhance the Vc content in tomato fruits [43]. These cross-crop consistencies indicate that the improvement of functional components by slow-release fertilizers is not accidental, but is closely related to the plant physiological responses induced by their unique nutrient supply patterns.

Photosynthesis is the primary driving force for yield formation and quality development, and its photosynthetic rate is widely recognized as one of the most reliable physiological indicators for crop growth and productivity [44]. The results of this study showed that the BCSRF treatment, especially the T3 treatment, maintained a significantly higher Pn than the control group throughout the period of leaf photosynthesis measurement (Figure 11A), while also increasing WUE (Figure 11C). This provided a sufficient nutritional foundation for fruit set and fruit cell expansion of Huizao, thereby significantly improving the yield per plant of Huizao (Table 1). Slow-release nitrogen fertilizer and nitrogen application rates significantly affect the physiological characteristics of sunflower crops; it was found that sunflower exhibited the highest leaf net photosynthetic rate, yield, and yield components when applied with neem-coated urea slow-release fertilizer [45]. Sulfur-coated urea (SCU) and resin-coated urea (RCU) continuously promoted the increase in Pn, which is conducive to increasing starch content and thus improving the yield of lotus leaves [46]. The above results are consistent with those of this study. However, there are differences: the BCSRF used in this study has different material components from neem-coated urea slow-release fertilizer, and BCSRF was applied only once, whereas SCU and RCU were applied in split applications during different growth stages of lotus leaves. Nevertheless, these studies collectively indicate that slow-release fertilizer technology can significantly improve the photosynthetic performance of crops, thereby providing a more sustained and efficient “source” driving force for yield formation.

It has been widely demonstrated that different types of slow/controlled-release fertilizers are effective in improving crop yield and quality [47,48,49]. However, there are significant differences in their action mechanisms, application conditions, and economic costs, which directly affect their application potential in fruit tree cultivation. In comparison, the bag-controlled slow-release fertilizer technology (e.g., BCSRF used in this study), as a physical controlled-release method, exhibits certain slow-release effects and applicability. Nevertheless, this technology is only a preliminary investigation on Huizao in southern Xinjiang, and more in-depth research is still needed regarding the economic evaluation such as cost–benefit analysis. Future research should further quantify its long-term agronomic benefits, economic benefits, and environmental impacts under different production conditions, so as to promote the standardized and industrialized application of this technology.

## 4. Materials and Methods

### 4.1. Plant Materials

The experiment was conducted from 2023 to 2024 at the southern Xinjiang (Jujube) Comprehensive Experimental Station of the National Peach Industry Technology System, situated in Maigaiti County, Kashgar Prefecture, Xinjiang Uygur Autonomous Region. The preparatory work, including site selection and variety tree selection, was completed in 2023, while the application of BCSRF, sample collection, and data analysis were completed in 2024.

This region has a typical arid continental climate, with abundant sunshine, large annual and daily temperature variations, and significant diurnal temperature differences. The jujube orchard at the experimental site has deep soil layers, which are categorized as sandy loam. It also boasts flat terrain, a neat orchard layout, and well-managed conditions.

**Figure 13:**
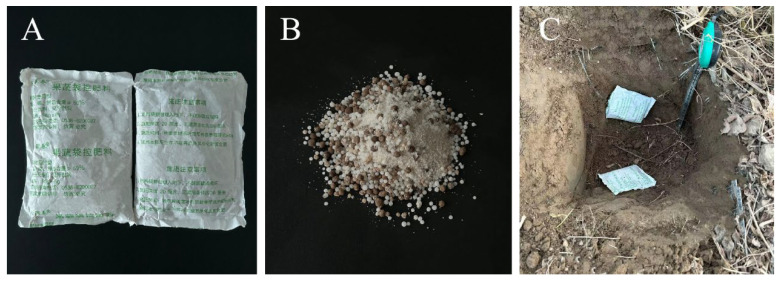
BCSRF and application depth. Note: (**A**) Outer packaging of BCSRF. (**B**) Internal components of BCSRF. (**C**) Application depth of BCSRF.

The jujube trees used in the experiment were Huizao trees, with a tree age of 16 years (planting spacing: 1.5 m × 4.0 m). The slow-release fertilizer tested had the following specifications: Each bag weighed 95 ± 8 g, with a release period of 180–210 days. The nutrient ratio of N/P_2_O_5_/K_2_O was 19:9:22, and the total nutrient content was ≥50% (Figure 13A,B).

### 4.2. Experimental Design

Under conventional jujube tree management conditions, four treatments were set up with no BCSRF application as the control: CK (0 kg/ha), T1 (22 kg/ha), T2 (44 kg/ha), and T3 (66 kg/ha). Jujube trees with basically consistent size and structure were selected, and a randomized block design was adopted, with 3 experimental plots set up. In each plot, each treatment had 3 replicates, with 20 jujube trees in each replicate, totaling 240 jujube trees. In each treatment, 3 jujube trees with uniform growth were selected as sample trees for all measurements, and the remaining jujube trees were used as guard rows. Other management measures were kept consistent across all treatments.

The BCSRF was applied on 22 March 2024 (before bud burst period). During fertilization, two parallel trenches were dug on each side of each row of fruit trees. The inner edge of each trench was 40 cm away from the trunk, with a width of 30–40 cm and a depth of 20 cm. The fertilizer bags were laid flat in the trenches on both sides of the plants, with a spacing of 5–10 cm between adjacent fertilizer bags (Figure 13C). After laying the bags, the fertilization trenches were filled and leveled with soil.

### 4.3. Measurement Indicators and Methods

#### 4.3.1. Determination of Soil Nutrient Indicators

Soil samples from the 0–20 cm, 20–40 cm, and 40–60 cm soil layers were collected using a soil auger. Samples from the same soil layer were thoroughly mixed, and approximately 1 kg of each mixed sample was retained using the quartering method. Impurities such as stones and residual plant roots were removed from the soil. After air-drying, the samples were sieved through 0.15 mm, 0.25 mm, and 2.00 mm sieves, respectively, and then placed into labeled self-sealing bags.

Soil alkali-hydrolysable nitrogen (AHN) was measured using the alkali hydrolysis diffusion. Soil available phosphorus (AP) was measured using the 0.5 mol/L NaHCO_3_ extraction-molybdenum antimony anti-colorimetry. Soil available potassium (AK) was measured using the 2 mol/L nitric acid extraction-inductively coupled plasma optical emission spectrometry (ICP-OES). Soil organic matter (OM) was calculated using the potassium dichromate volumetric method. All of the above-mentioned investigations referred to the Soil Agrochemical Analysis, 3rd Edition [50].

#### 4.3.2. Observation of Phenological Period and Determination of Growth Indicators and Yield Indicators

In 2024, phenological observations were conducted, covering the bud burst period, leaf expanding period, initial flowering period, full flowering period, final flowering period, fruit white ripening period (WR), fruit crisp ripening period (CR), fruit full ripening period (FR), and leaf fall period. Meanwhile, growth indicators were determined. For each treatment, 3 trees were selected for investigation. From each tree, 7 short bearing branches, 5 annual branches, and 5 secondary branches were chosen, and their lengths and thicknesses were measured using a standard tape measure. The leaf length, leaf width, and leaf area were measured with a Wanshen leaf area meter (Wanshen, Hangzhou Wanshen Electronic Technology Co., Ltd., Hangzhou, China).

Additionally, 10 representative biennial branches with relatively consistent growth (each branch was approximately 80–100 cm in length and had 5–8 nodes) were selected. From 23 to 24 May 2024, the number of flowers per inflorescence and the number of flowers per short bearing branch were investigated, and the flower thickness was measured. On 12 July 2024, the fruit set rate was surveyed. The calculation formula for the fruit set rate was as follows: Fruit set rate (%) = (Number of fruits set on sample branches/Number of inflorescences on sample branches) × 100%. The average fruit set rate of the sample plants was regarded as the fruit set rate (%) of the experimental plot.

On 3 November 2024, the yield per plant was determined. The yield per plant was weighed using an electronic scale, and the average yield per plant from 3 replicates was taken as the yield per plant of the treatment. All of the above-mentioned investigations referred to the Descriptors and Data Standard for Jujube (*Ziziphus jujuba* Mill.) [51].

#### 4.3.3. Determination of Fruit Quality Indicators

Fruits were collected at three stages: WR, CR, and FR. For each treatment, jujube fruits were randomly collected from the sample trees along the four main directions (east, west, south, and north) around the crown. A total of 90 fruits were collected for each treatment. After picking, the fruits were put into prepared sampling bags, labeled properly, and then placed in a sampling box and taken back to the laboratory. Thirty randomly selected Huizao fruits were regarded as one replicate, with three replicates in total. The jujube fruits were sliced, the flesh was frozen in liquid nitrogen and then ground into powder, and the sample powder was stored in a −80 °C refrigerator for subsequent determination of quality indicators.

Determination of fruit appearance quality: Fruit weight: The weight of a single fruit was measured using an electronic balance. Fruit shape: The transverse thickness and longitudinal thickness of the fruit were measured with an electronic vernier caliper. The fruit shape index was calculated according to the formula: Fruit shape index = Longitudinal thickness of fruit/Transverse thickness of fruit.

Determination of fruit nutritional quality: Soluble sugar (SS) was measured using the anthrone colorimetry method; titratable acid (TA) was measured using the 0.1 mol/L NaOH standard solution titration method; reduced vitamin C (Vc) was titrated with 2,6-dichloroindophenol; total flavonoids (TF) were determined using NaNO_2_-Al(NO_3_)_3_ spectrophotometry; total phenols (TP) were determined using Folin–Ciocalteu reagent; cyclic adenosine monophosphate (cAMP) and cyclic guanosine monophosphate (cGMP) were determined using high-performance liquid chromatography (HPLC). All of the above-mentioned investigations referred to the Plant Physiology Laboratory Manual [52].

#### 4.3.4. Determination of Photosynthetic Indicators

Photosynthetic parameters: Pn—net photosynthetic rate, Gs—stomatal conductance, Ci—intercellular carbon dioxide concentration, Tr—transpiration rate, WUEp—water use efficiency, and the formula is WUEp = Pn/Tr. The measurement of photosynthetic indexes was carried out at 125 d, 154 d, and 186 d after the application of BCSRF. The measurement was carried out using a TARGA-1 photosynthetic instrument (PPSystems, Haverhill, MA, USA) from 10:00 to 13:00 on sunny days. The photosynthetically active radiation intensity was maintained at 2000 μmol/(m^2^·s) using the LED light source built into the instrument, the module temperature was set at 25 °C, the CO_2_ reference concentration was set at 375 μmol/mol, and a constant air flow rate of 400 μmol/s was maintained with the gas path kept open. The readings were recorded after they stabilized. Three healthy sample plants with uniform growth were selected, and the sun-exposed functional leaves (the 4th leaf counted from the base of the jujube bearing shoot) on the current-year jujube bearing shoots with consistent growth status and branch length in four different directions (east, south, west, and north) of the tree were selected for measurement, with five leaves measured for each plant. All of the above-mentioned investigations referred to the method described by An et al. [53].

### 4.4. Data Processing and Analysis

Excel 2021 (Microsoft Corporation, Redmond, WA, USA)was used to process the data. A one-way analysis of variance (ANOVA) test for the data was conducted, and the significant differences among them were compared with a Duncan’s test (*p* < 0.05) using SPSS 27.0 (SPSS, Inc., Chicago, IL, USA), and Origin 2017 (OriginLab, Northampton, MA, USA.) was used to map.

## 5. Conclusions

The BCSRF exhibits a certain slow-release effect in southern Xinjiang and, to a certain extent, promotes the coordinated improvement of soil nutrient content, Huizao yield, and quality. Among all treatments, the T3 treatment showed the best effect: it increased the contents of AHN, AP, and AK of BCSRF at the four sampling points and also enhanced the contents of soluble sugar, cAMP, and cGMP in Huizao fruits. The results of this study provide a scientific basis for the development of slow-release fertilizers suitable for Huizao in southern Xinjiang and offer a reference for optimizing fertilization schemes of other field crops.

## Figures and Tables

**Table 1:** Effects of different BCSRF treatments on floral traits, fruit setting rate, and yield of Huizao.

Treatment	Flower Diameter Size	Number of Flowers per Inflorescence	Number of Flowers Formed on Jujube Hanging Branches	Fruit Setting Rate (%)	Yield per Plant (kg/Plant)
CK	0.78 ± 0.04 a	11.88 ± 0.45 a	36.44 ± 0.04 a	12.21 ± 0.98 b	20.97 ± 0.47 c
T1	0.79 ± 0.01 a	11.93 ± 0.67 a	37.94 ± 6.09 a	14.37 ± 1.12 ab	24.16 ± 0.40 b
T2	0.81 ± 0.02 a	11.94 ± 0.37 a	42.64 ± 14.17 a	14.59 ± 0.93 ab	24.60 ± 1.08 b
T3	0.81 ± 0.06 a	12.07 ± 0.61 a	52.33 ± 4.76 a	15.10 ± 2.24 a	29.22 ± 3.00 a

Note: Different lowercase letters in the same column indicate a significant difference between treatments (*p* < 0.05).

**Table 2:** Effects of different BCSRF treatments on fruit weight and shape dimensions of Huizao at different ripening stages.

Treatment	Single Fruit Weight (g)	Longitudinal Thickness (mm)	Transverse Thickness (mm)	Fruit Shape Index	Period
CK	10.32 ± 0.72 b	35.92 ± 0.25 b	24.50 ± 1.35 a	1.48 ± 0.09 a	
T1	10.59 ± 0.80 b	37.67 ± 0.85 a	25.36 ± 2.50 a	1.50 ± 0.14 a	White ripening
T2	11.24 ± 0.28 b	37.89 ± 0.41 a	25.42 ± 2.69 a	1.51 ± 0.16 a	
T3	13.14 ± 0.23 a	38.78 ± 0.91 a	26.97 ± 1.55 a	1.45 ± 0.11 a	
CK	11.17 ± 1.72 a	37.38 ± 2.75 a	25.34 ± 3.67 a	1.51 ± 0.14 a	
T1	12.06 ± 1.54 a	37.60 ± 4.27 a	26.31 ± 3.41 a	1.45 ± 0.07 a	Crisp ripening
T2	12.85 ± 1.58 a	38.61 ± 2.38 a	26.65 ± 3.27 a	1.46 ± 0.09 a	
T3	13.82 ± 0.64 a	39.06 ± 1.69 a	26.95 ± 0.58 a	1.47 ± 0.09 a	
CK	12.18 ± 0.51 b	38.12 ± 0.35 a	28.68 ± 1.83 a	1.43 ± 0.12 a	
T1	12.47 ± 0.09 b	38.79 ± 1.62 a	30.29 ± 2.09 a	1.36 ± 0.11 a	Full ripening
T2	13.39 ± 0.31 a	38.80 ± 1.90 a	30.51 ± 1.07 a	1.37 ± 0.84 a	
T3	13.96 ± 0.32 a	38.90 ± 0.66 a	30.56 ± 1.59 a	1.48 ± 0.17 a	

Note: Different lowercase letters in the same column indicate a significant difference between treatments (*p* < 0.05).

## Data Availability

The original contributions presented in this study are included in the article. Further inquiries can be directed to the corresponding authors.
